# Gut microbiota and inflammasome-mediated pyroptosis: a bibliometric analysis from 2014 to 2023

**DOI:** 10.3389/fmicb.2024.1413490

**Published:** 2025-01-06

**Authors:** Hang Zhang, Tian Zhao, Juan Gu, Fushan Tang, Lei Zhu

**Affiliations:** ^1^Department of Clinical Pharmacy, Key Laboratory of Basic Pharmacology of Guizhou Province and School of Pharmacy, Zunyi Medical University, Zunyi, China; ^2^Key Laboratory of Basic Pharmacology of Ministry of Education and Joint International Research Laboratory of Ethnomedicine of Ministry of Education, Zunyi Medical University, Zunyi, China; ^3^The Key Laboratory of Clinical Pharmacy of Zunyi City, Zunyi Medical University, Zunyi, China; ^4^Department of Pharmacy, Affiliated Hospital of Zunyi Medical University, Zunyi, Guizhou, China

**Keywords:** bibliometrics, gut microbiota, inflammasome, pyroptosis, butyrate, inflammatory diseases

## Abstract

**Background:**

The role of gut microbiota in inflammatory disease development and progression has been recognized more recently. Inflammasome-mediated pyroptosis in involved in these diseases. This complex relationship between gut microbiota and inflammasome-mediated pyroptosis provides an important field of research. Bibliometric analysis provides a comprehensive understanding of this relationship, offering valuable insights into emerging research trends.

**Materials and methods:**

Leveraging data spanning from 2014 to 2023 sourced from the Web of Science Core Collection, our analysis was conducted using advanced tools such as SCImago Graphica, VOSviewer, and CiteSpace software. Visualizations were created using GraphPad Prism software. We explored the nuanced aspects of research hotspots, collaborative networks, and developing trends in this field.

**Results:**

A global bibliometric analysis identified 520 relevant studies spanning 41 countries and 887 institutions. Over the past decade, publication trends have shown consistent growth, with China and the United States leading the research output. Southern Medical University and Nanjing Medical University in China emerged as leading institutions in this filed. Prominent contributors include Jia Sun, Yuan Zhang, Wei Chen, Jing Wang, and Hongtao Liu from China, alongside Eicke Latz from Germany. High-impact journals such as *Frontiers in Immunology* and *Nature Communications* have been pivotal in disseminating research in this domain. Keyword analysis highlighted a primary focus on gut microbiota, NLRP3 inflammasome, pyroptosis pathways, and inflammatory diseases, themes that persist in recent studies. Furthermore, burst keyword analysis identified “butyrate” as the sole term currently experiencing a marked increase in research interest.

**Conclusion:**

Research has been deeply focused on the gut microbiota and inflammasome triggered pyroptosis in years. Over the past decade, the exploration of how gut microbiota and NLRP3 or NLRP6 inflammasome-mediated pyroptosis has been an area of interest. Future investigations in this filed may primarily revolve around understanding the correlation between butyrate and NLRP3 inflammasome induced pyroptosis in relation to conditions. However, an in-depth analysis, through studies is crucial to uncover and elucidate the complex mechanisms linking these elements.

## 1 Introduction

The intestinal tract harbors a diverse community of microbiota comprising tens of trillions of microorganisms, including bacteria, viruses, fungi, and protozoans (Pellegrini et al., 2020). In addition to supporting fundamental physiological functions, the gut microbiota plays a crucial role in maintaining intestinal homeostasis that is regulated by intricate interactions between the host immune system, gut microbiota, and the intestinal epithelial barrier. Disruption of this delicate balance can precipitate various inflammatory conditions, leading to dysbiosis (structural and metabolic alterations in the microbiota), damage to the intestinal barrier, and initiation of aberrant immune responses (Lei-Leston et al., 2017; Ji et al., 2023). Recent advancements in 16S rRNA sequencing technology have highlighted the complex relationship between the gut microbiota and various diseases (Wang S. et al., 2023). The alterations in the gut microbiota can influence the development of cardiovascular diseases (Wang et al., 2022), neurodegenerative disorders (Sun et al., 2021), and metabolic diseases (Shi et al., 2022).

Pyroptosis is a programmed cell death pathway characterized by chromatin condensation, nuclear integrity, cell swelling, and plasma membrane rupture (Huang et al., 2021). Alterations in the gut microbiota are closely associated with the development of pyroptosis. Dysbiosis leads to an increase in pathogen-associated molecular patterns (PAMPs) within the gut, which subsequently activate inflammasome-mediated pyroptosis. This process ultimately results in the onset of inflammatory lesions in the gut (Xue et al., 2022). Inflammasome-mediated pyroptosis refers to the process by which pattern recognition receptors (PRRs), located within cells or on the cell membrane, recognize PAMPs or damage-associated molecular patterns. Upon recognition, PRRs interact with caspase-1, either with or without the co-binding of ASC. This assembly triggers the activation of caspase-1 and promotes the transcription and expression of inflammatory cytokines interleukin 1β (IL-1β) and IL-18. Activated caspase-1 cleaves gasdermin D (GSDMD) to release its active N-terminal domain. This domain forms pores in the cell membrane and initiates pyroptosis and inflammatory responses (Zheng et al., 2020; Liu et al., 2023; Wang J. et al., 2023). Excessive or uncontrolled pyroptosis has been implicated in the development of various diseases, including cardiovascular, liver, and neurological diseases, as well as other inflammatory disorders (Rao et al., 2022). These findings highlight the therapeutic potential of targeting and inhibiting inflammasome-mediated pyroptosis. Moreover, emerging evidence suggests that the interaction between the gut microbiota and inflammasome-mediated pyroptosis plays an important role in regulating gut homeostasis (Lei-Leston et al., 2017). Understanding this relationship is essential for unveiling the role of gut microbiota in human diseases and could guide the development of novel therapeutic strategies.

Bibliometrics, a method for quantitatively analyzing a research field based on textual data (Wang Y. et al., 2023), has gained prominence since its establishment as an independent discipline in 1969, and it is widely accepted as scientifically credible (Chen et al., 2022). Researchers have extensively employed bibliometrics to identify current hotspots within their field of study, track the most frequently cited publications and articles, and forecast future research trends (Zhang B. et al., 2023). A notable increase in the number of publications focused on gut microbiota and inflammasome-mediated pyroptosis has been reported. Investigating the intricate interconnection between the two entities can yield profound insights into their influential roles within the context of disease. In this study, we performed a bibliometric analysis of the literature examining the gut microbiota and pyroptosis mediated by inflammasomes from 2014 to 2023. Initially, we employed a visualization software to scrutinize and present data regarding countries, institutions, authors, journals, co-cited literature, and keywords. Subsequently, we discussed and predicted past research hotspots and future research trends. Furthermore, we summarize the mechanistic relationship between butyrate and NLRP3 inflammasome-mediated pyroptosis. This study provides new perspectives for researchers in this field, enabling them to broaden their research scope.

## 2 Materials and methods

### 2.1 Data sources and search strategy

**FIGURE 1:**
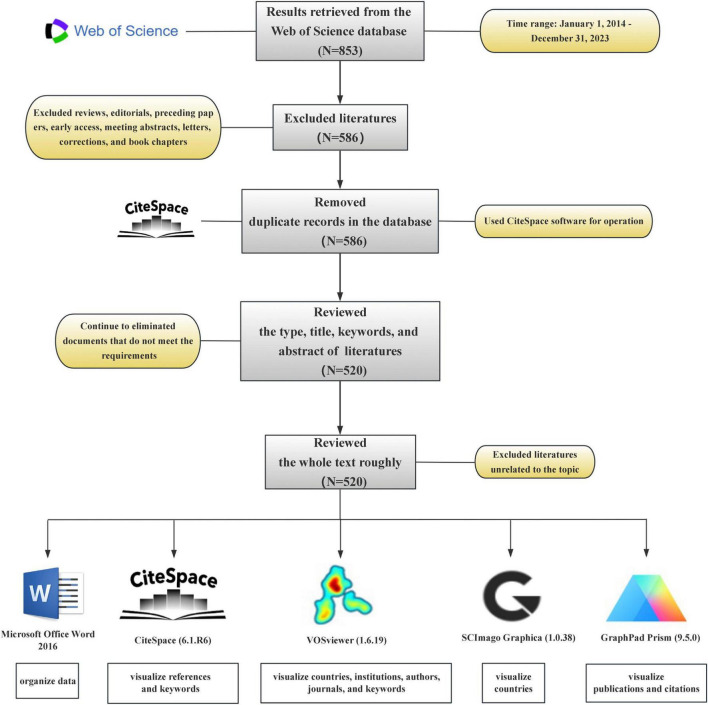
Flowchart of the document retrieval process and research software.

The Web of Science database is an important resource for accessing academic information and encompasses many authoritative journals worldwide. Data used in this study were obtained from the Web of Science Core Collection. In the first step, to enhance the comprehensiveness of the search strategy, we refined the strategy to include: ([TS = “Intestinal flora” OR “Intestinal microflora” OR “Gut flora” OR “Bowel flora” OR “Gut microbiome” OR “Gut microbiota”] AND [TS = “Pyroptosis” OR “Inflammasome?” OR “NLRP1” OR “NLRP2” OR “NLRP3” OR “NLRP6” OR “NLRP7” OR “NLRP12” OR “NLRC4” OR “AIM2” OR “Pyrin” OR “IFI16”]) for the period from 1 January 2014 to 31 December 2023 followed by a web-based search. Second, literature types other than research papers were excluded from the left interface of the Web of Science. In the third step, the complete dataset generated was exported as a text file, and this was followed by deduplication using the CiteSpace software. In the fourth step, the literature types, titles, keywords, and abstracts within the text file were read to determine if they met the requirements. Documents that did not meet the requirements were excluded. In the fifth step, a general read-through of the entire text was conducted to determine whether it aligned with theme requirements, and those that did not were removed. Finally, 520 studies that met these requirements were identified (Figure 1).

### 2.2 Data analysis and visualization

VOSviewer^1^ is a robust software tool for bibliometric visualization that offers a range of functionalities, including network, coverage, and density visualization. These capabilities provide valuable insights into collaborative relationships, research hotspots, and emerging trends in specific academic fields (Huang et al., 2024). VOSviewer (version 1.6.19) software was used for the statistical analysis and visualization of countries, institutions, authors, journals, and keywords. The VOSviewer parameters were configured as follows: full counting was used; the threshold values were determined based on the specific objects being analyzed.

CiteSpace^2^ is a knowledge visualization tool developed based on co-citation analysis that facilitates the visualization of citation patterns, collaborative relationships, and shifts in research hotspots (Song et al., 2023; Wang J. et al., 2023). CiteSpace software (version 6.1.R6) was used to statistically analyze the co-citation literature and keywords. The parameters for CiteSpace were configured as follows: time span (January 2014 to December 2023); years per slice (1); selection criteria (Top N: top 50); link strength (cosine; scope: within slices).

SCImago Graphica^3^ is a software application designed to generate visualizations that depict the geographic distribution of literature at the national level (Zhang et al., 2022). The data for SCImago Graphica were sourced directly from GML files exported from VOSviewer. This study primarily used the SCImago Graphica (version 1.0.38) software to visualize the published literature by country.

GraphPad Prism software (version 9.5.0)^4^ was used to visualize annual publication and citation volumes over the course of the study period. Microsoft Office Word 2016 (pre-installed software) was used to systematically organize and structure the relevant data.

## 3 Results

### 3.1 Basic information about the documents and citations

**FIGURE 2:**
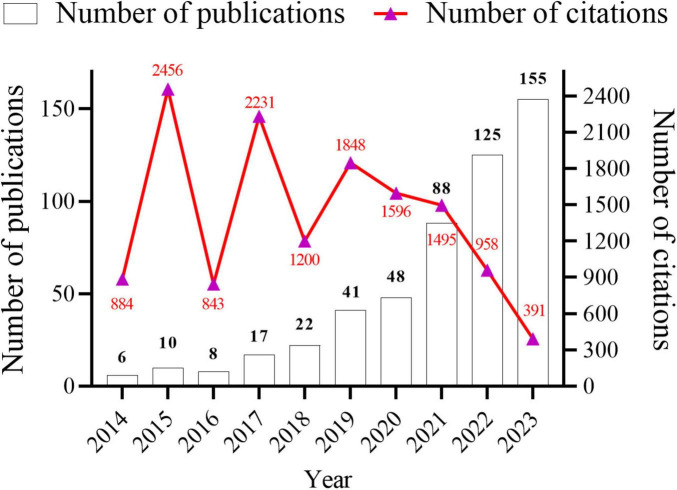
Annual number articles published and citations from January 2014 to December 2023 in gut microbiota and inflammasome-mediated pyroptosis.

Data analysis was conducted using both CiteSpace and VOSviewer software. A total of 520 research papers originating from 41 countries and 887 institutions with 4,212 authors were analyzed. These publications were dispersed across 245 journals, collectively citing 18,973 articles from 2,751 journals. The temporal trend graph of specific publications and citation counts from 2014 to 2023 is presented in Figure 2. The annual number of published citations exhibited an explosive increase in 2015 and 2017, with citations exceeding 2,000. This is attributable to the emergence of three highly cited articles in 2015 and 2017, each garnering more than 600 citations. The steady increase from 6 publications in 2014 to 155 in 2023 indicates a consistent interest from researchers in this field. However, the number of citations in 2023 decreased by nearly half compared to that in 2022, likely because the papers have just begun to be published, and it will take some time for the number of citations to rise. In summary, the overall trend in publications over the past decade has been increasing, with the number of articles published in recent years being more than 10-fold higher than that published in 2014, suggesting that this field continues to garner the attention of researchers and is steadily advancing.

### 3.2 Contributions of countries and institutions

To investigate which countries and institutions worldwide have made notable contributions to the field under study, publications from 41 countries and 887 institutions worldwide were analyzed. Data from Taiwan and China were consolidated into calculations for China.

**FIGURE 3:**
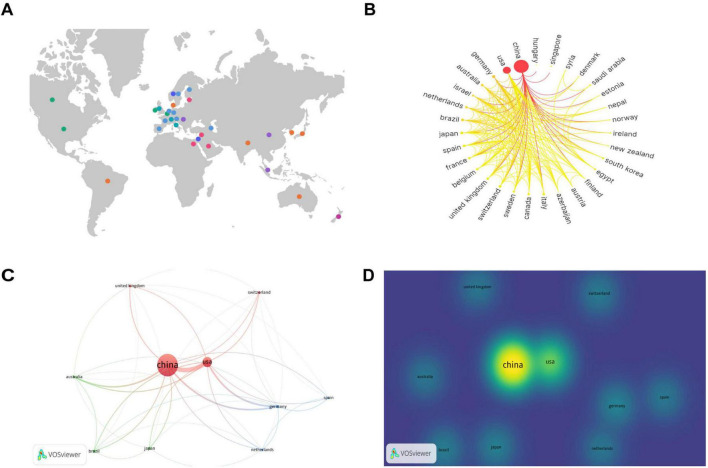
Contributions and relationships with major countries. **(A)** Global distribution map of countries involved in the research topic. **(B)** Circular map of countries contributing to the subject of this study. **(C)** Network visualization of the top 10 countries in terms of the number of publications. **(D)** Density visualization of the top 10 countries in terms of the number of publications.

To analyze the contributions by country, a knowledge graph visualization of 41 countries worldwide was initially conducted using SCImago Graphica software. Figure 3A indicates the broad distribution of nations engaged in the research field under study, predominantly concentrated in the North American, European, Middle Eastern, Eastern Asian, and Oceanian regions. Researchers from these countries in these regions have attracted considerable interest in the field.

The number of publications per country is indicated on the circular map in Figure 3B. A larger outermost circular area signifies a higher number of publications in that country. The gradient color scheme divides the figure into seven stages, with darker colors from left to right indicating a greater number of publications and closer collaboration among different countries. The analysis indicates that countries on the left side of the circular ring produced the highest number of publications and collaborations, and a considerable proportion of these were developed nations. China and the United States exhibit the highest number of publications and close collaboration with researchers from other countries.

Subsequently, we conducted a further analysis of the top 10 countries with the highest number of articles in the field of study. Table 1 indicates that China produced the highest number of publications, with 364 articles substantially contributing to the field and accounting for 63.64% of the total number of publications in the top 10 countries. Additionally, the United States yielded the highest number of citations, with 5,841 citations, indicating its substantial contribution to this field. From an average citation perspective, Germany, Switzerland, Australia, and United Kingdom each exhibited a mean of more than 100 citations per article, suggesting that although these countries had fewer publications, the quality of their publications was high.

**TABLE 1:** Top 10 countries with the most publications.

Rank	Country	Number of articles	Number of citations	Mean citations per article
1	China	364	5,384	14.79
2	United States	104	5,841	56.16
3	Germany	17	1,867	109.28
4	Japan	15	1,406	93.73
5	Brazil	14	1,159	82.78
6	Switzerland	13	1,423	149.46
7	Spain	12	570	47.50
8	Australia	11	1,553	141.18
9	Netherlands	11	522	47.45
10	United Kingdom	11	1,362	123.82

The top 10 countries with the most publications were then visually analyzed using VOSviewer software. Figure 3C provides a network visualization, where a larger circular area indicates a higher volume of publications, thicker connections between nodes indicate a greater number of collaborative publications between the two countries, and node color represents different clusters. Figure 3D provides a density visualization, and a deeper yellow fluorescence corresponds to a higher number of publications. From the analysis of Figures 3C, D, it can also be observed that among the top 10 countries by number of publications, China exhibits the highest number of publications, and the United States, Germany, and Australia collaborate most closely with the other top 10 countries in terms of the number of publications.

Academic institutions that made outstanding contributions to the field were analyzed using VOSviewer software and are summarized in Table 2. It can be observed that the top 10 institutions in terms of the number of publications were all universities from China. Among them, Southern Medical University, with 15 publications, ranked top in terms of the number of publications, whereas Nanjing Medical University, with 206 citations and an average citation rate of 20.60, ranked first in terms of the number of citations.

**TABLE 2:** Top 10 core research institutions with the most publications.

Rank	Organization (country)	Number of articles	Number of citations	Mean citations per article
1	Southern Medical University (China)	15	199	13.26
2	Soochow University (China)	13	107	8.23
3	Sichuan University (China)	12	175	14.58
4	Shandong University (China)	12	128	10.66
5	China Agricultural University (China)	11	130	11.82
6	Nanjing Medical University (China)	10	206	20.60
7	Zhejiang University (China)	10	148	14.80
8	Chinese Academy of Sciences (China)	9	41	4.56
9	Jilin University (China)	9	113	12.56
10	Nanjing University of Chinese Medicine (China)	9	89	9.88

**FIGURE 4:**
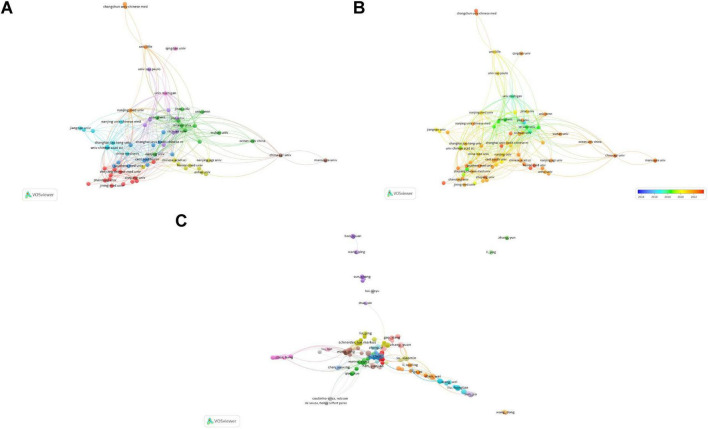
Contributions and relationships with major institutions and authors. **(A)** Network visualization of institutional collaboration. **(B)** Overlay visualization of institutional collaboration. **(C)** Network visualization of core author collaboration.

Subsequently, institutions with more than four publications were analyzed. Figure 4A presents the network visualization and collaborations between research institutions. A larger circular area indicates a higher volume of publications, and a thicker line connecting two institutions indicates closer collaboration. A total of 70 nodes and 9 clusters were obtained, but only 69 nodes were observed to collaborate with each other to the figure. These 69 institutions formed 9 clusters, with active cooperation among institutions within the same cluster. Yale University exhibits the most intense academic collaboration with other institutions.

Figure 4B presents an overlay visualization of research trends by institution. It can be used to analyze changes in research trends. Deep blue represents the earliest research institutions, and deep red indicates institutions that began publishing research most recently. It is evident that the majority of institutions have emerged as leading research institutions only in the past few years.

Based on the above analysis, cooperation among institutions from different countries and regions must be strengthened to promote better development in this field.

### 3.3 Contributions of authors and journals

According to the analysis using VOSviewer software, a total of 4,212 authors participated in the field of study during the study period. The minimum publication count for a core author was calculated based on Price’s Law (Hua and Wu, 2021), with a minimum publication count *m* = 0.749 × $\sqrt{n_{m\,a\,x}}$ ≈ 1.675 (where *n*_*max*_ represents the maximum publication count of an individual author). Therefore, authors who published two or more articles were identified as core authors in this field, with 465 core authors recorded, accounting for approximately 11.04% of the total number of authors. Table 3 indicates the top 10 core authors with the most publications, with Jia Sun, Yuan Zhang, Wei Chen, Jing Wang, and Hongtao Liu from China ranking first with five publications each. Eicke Latz from the University of Bonn in Germany ranked first in terms of author impact with a total citation count of 725 and an average citation count of 181.25. He has been recognized by the Web of Science as a “Highly Cited Researcher in the field of Immunology” annually since 2014. Additionally, he published a groundbreaking article titled “Microbiota-modulated metabolites shape the intestinal microenvironment by regulating NLRP6 inflammasome signaling” in *Cell* that boasts an impact factor (IF) of 64.5 on 3 December 2015.

**TABLE 3:** Core authors with the top 10 most publications.

Rank	Author	Number of articles	Number of citations	Mean citations per article
1	Jia Sun (China)	5	187	37.40
2	Yuan Zhang (China)	5	155	31.00
3	Wei Chen (China)	5	75	15.00
4	Jing Wang (China)	5	54	10.80
5	Hongtao Liu (China)	5	17	3.40
6	Eicke Latz (Germany)	4	725	181.25
7	Kai Markus Schneider (United States)	4	204	51.00
8	Christian Trautwein (Germany)	4	204	51.00
9	Jiahong Li (China)	4	159	39.75
10	Yu Wang (China)	4	147	36.75

Collaborative relationships among the 465 core authors are presented in Figure 4C. Of the 465 core authors, 358 collaborated with other authors. These 358 authors were categorized into 21 clusters. Although the connections between different clusters were relatively loose, collaboration between two interconnected clusters was present to a certain extent. This suggests that cooperation among authors conducting research in this field is not yet mature and there is a need to further enhance academic exchange and collaboration. Based on the analysis presented in this subsection, researchers can seek future collaboration and support from authors or teams with high publication volumes and impact factors.

Analysis of journals can assist in keeping pace with current research directions and hotspots. Between 2014 and 2023, 245 journals published articles in this field. Of the top 10 journals in terms of publication volume (Table 4), all were published in the Q1. *Frontiers in Immunology* ranked first in terms of the number of publications, with 19 publications. *Nature Communications*, a multidisciplinary journal that publishes high-quality research from various natural science fields, ranked first in terms of the number of citations, with 1,180 citations and an average citation rate of 147.50 per article. This journal has made a substantive contribution to advancements in this field. This analysis enables researchers to focus on the top 10 core journals to track hotspots and keep up with the latest research progress.

**TABLE 4:** Core journals with the top 10 most publications.

Rank	Source	Number of articles	Number of citations	Mean citations per article	Impact factor (2023–2024)	Category quartile (CiteScore)
1	*Frontiers in Immunology* (Switzerland)	19	252	13.26	5.7	Q1
2	*Journal of Agricultural and Food Chemistry* (United States)	16	168	10.50	5.7	Q1
3	*Food & Function* (United Kingdom)	16	237	14.81	5.1	Q1
4	*Frontiers in Pharmacology* (Switzerland)	13	144	11.08	4.4	Q1
5	*Journal of Ethnopharmacology* (Ireland)	11	121	11.00	4.8	Q1
6	*International Journal of Molecular Science* (United States)	10	106	10.60	4.9	Q1
7	*Molecular Nutrition & Food Research* (Germany)	10	172	17.20	4.5	Q1
8	*Biomedicine & Pharmacotherapy* (France)	9	47	5.22	6.9	Q1
9	*Nature Communications* (United Kingdom)	8	1,180	147.50	14.7	Q1
10	*Gut Microbes* (United States)	8	139	17.38	12.2	Q1

### 3.4 Co-citation of references and references with citation bursts

When two or more articles are simultaneously cited in a single work, the relationship between them is termed co-citation. A closer co-citation relationship indicates a high degree of similarity in content among the cited papers (Wang S. et al., 2023). Table 5 presents the top 10 co-cited references ranked by frequency. Their publication dates were primarily between 2010 and 2015, with research primarily focusing on NLRP3, NLRP6, and gut microbiota. The most frequently co-cited article was ‘NLRP6 inflammasome is a regulator of colonic microbial ecology and risk for colitis’ published in Cell (Elinav et al., 2011). This study reveals the regulatory role of the NLRP6 inflammasome in colonic microbiota and colitis, providing a novel mechanism and target for a deeper understanding of the relationship between colonic microbiota and colitis. The most highly cited article was “Microbiota-Modulated Metabolites Shape the Intestinal Microenvironment by Regulating NLRP6 Inflammasome Signaling” (Levy et al., 2015) that elucidated the regulatory role of intestinal microbial metabolites on the NLRP6 inflammasome signaling pathway, clarified the interaction mechanism between intestinal microbes and the host immune system, and provided new strategies for treating intestinal inflammatory diseases. The NLRP6 inflammasome is widely distributed across intestinal tissues and plays a role in disease progression by modulating the composition of the intestinal microbiome and integrity of the intestinal mucosal barrier (Nie and Zhu, 2021). Consequently, targeting NLRP6-mediated pyroptosis to address intestinal microbial imbalances may offer new avenues for clinical research and the treatment of diseases associated with these imbalances. NLRP6 inflammasome activation regulates gut microbiota through various mechanisms (Angosto-Bazarra et al., 2022). In this study, we focused on the pyroptosis pathway mediated by the NLRP6 inflammasome. Activation of the NLRP6 inflammasome can be modulated by microbial signals such as type I interferons (IFNs), whereas microbial components such as metabolites, RNA, lipoteichoic acid, and LPS can directly interact with the NLRP6 protein. This interaction induces inflammasome-mediated pyroptosis and regulates gut microbiota by promoting antimicrobial peptide secretion (Li and Zhu, 2020). It is important to note that there is an ongoing debate regarding whether the NLRP6 inflammasome activates the non-canonical pyroptosis pathway mediated by caspase-11 or the canonical pyroptosis pathway mediated by caspase-1 (Levy et al., 2017; Angosto-Bazarra et al., 2022; Li R. et al., 2022). Additionally, metabolites from the gut microbiota play a significant role in host immunity and microbial communication. For example, short-chain fatty acids stimulate the expression of NLRP6 inflammasomes in the gut, whereas taurine and histamine reduce their expression (Nie and Zhu, 2021). Given the complexity of gut microbiota composition, further research is needed to understand the effects of different types of microbiota metabolites on NLRP6 inflammasome-mediated pyroptosis and to explore the therapeutic potential of these metabolites.

**TABLE 5:** Top 10 co-cited references in the field.

Rank	Reference title	Journal	Co-citations	Year of publication	Reference
1	NLRP6 inflammasome is a regulator of colonic microbial ecology and risk for colitis	*Cell*	59	2011	Elinav et al., 2011
2	Microbiota-modulated metabolites shape the intestinal microenvironment by regulating NLRP6 inflammasome signaling	*Cell*	35	2015	Levy et al., 2015
3	NLRP6 inflammasome orchestrates the colonic host-microbial interface by regulating goblet cell mucus secretion	*Cell*	30	2014	Wlodarska et al., 2014
4	Metagenomic biomarker discovery and explanation	*Genome Biol*	28	2011	Segata et al., 2011
5	QIIME allows analysis of high-throughput community sequencing data	*Nat Methods*	28	2010	Caporaso et al., 2010
6	Colitis induced in mice with dextran sulfate sodium (DSS) is mediated by the NLRP3 inflammasome	*Gut*	28	2010	Bauer et al., 2010
7	The NLRP3 inflammasome protects against loss of epithelial integrity and mortality during experimental colitis	*Immunity*	27	2010	Zaki et al., 2010
8	Inflammasome-mediated dysbiosis regulates progression of NAFLD and obesity	*Nature*	24	2012	Henao-Mejia et al., 2012
9	UPARSE: highly accurate OTU sequences from microbial amplicon reads	*Nat Methods*	22	2013	Edgar, 2013
10	Cross-talk between *Akkermansia muciniphila* and intestinal epithelium controls diet-induced obesity	*Proc Natl Acad Sci U S A*	19	2013	Everard et al., 2013

**FIGURE 5:**
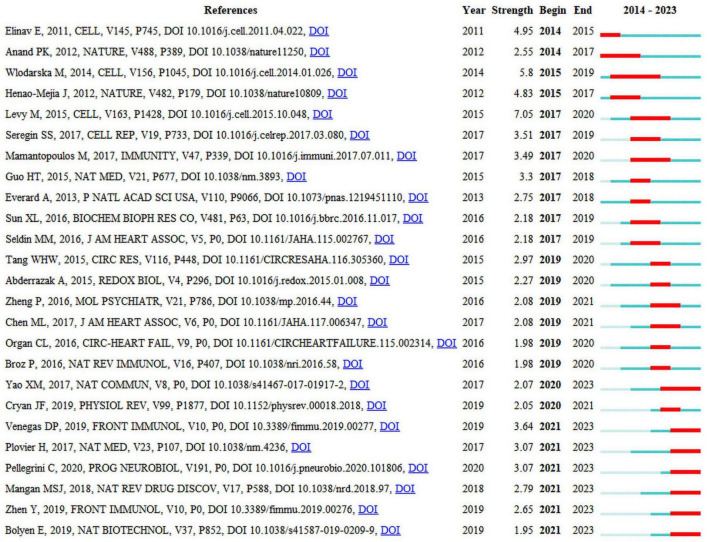
Top 25 references with the strongest citation bursts.

Seven of the 25 publications that are presented in Figure 5 will remain in a state of citation bursts in 2023 (Plovier et al., 2017; Yao et al., 2017; Mangan et al., 2018; Bolyen et al., 2019; Parada Venegas et al., 2019; Zhen and Zhang, 2019; Pellegrini et al., 2020). Strength indicates citation burst strength. The red and blue lines depict the citation burst from 2012 to 2023, and the length of the red line indicates the duration of the citation burst. From the highly cited publications, it can be concluded that researchers in this field are currently primarily focused on the interplay between the gut microbiota, NLRP3 inflammasome, and inflammatory diseases. Numerous studies have summarized the role of NLRP3 inflammasome-mediated pyroptosis in various diseases, including cardiovascular, neurological, respiratory, hepatic, renal, intestinal, metabolic, autoimmune, and neoplastic disorders (Tao et al., 2020; Liu et al., 2023; Shi and Xu, 2023). The activation or inhibition of NLRP3 inflammasome-mediated pyroptosis is beneficial for the treatment of these diseases. A bidirectional interaction exists between gut microbiota and the NLRP3 inflammasome, in which alterations in gut microbiota can lead to activation of the NLRP3 inflammasome, and activation of the NLRP3 inflammasome can also influence the composition and function of the gut microbiota. This interaction may occur through multiple pathways (Pellegrini et al., 2020). First, after being recognized by immune cells and intestinal epithelial cells, the gut microbiota activates the intracellular NLRP3 inflammasome, subsequently inducing the release of IL-18 that promotes the release of antimicrobial peptides, thereby regulating the balance between gut microbiota. Second, the gut microbiota promote the activation and assembly of the NLRP3 inflammasome by producing metabolites such as short-chain fatty acids (SCFAs) that subsequently promote the release of IL-18, thereby maintaining the integrity of the intestinal epithelial barrier. Third, the activated NLRP3 inflammasome promotes transformation of the gut microbiota into an anti-inflammatory phenotype, subsequently inducing T-cell maturation and differentiation for immune regulation. However, the current perspectives on the role of the NLRP3 inflammasome in regulating the intestinal microbiota are sharply divided (Nie and Zhu, 2020), suggesting that activation of the NLRP3 inflammasome facilitates the regulation of the intestinal microbiota to thereby ameliorate diseases, whereas others contend that activation of the NLRP3 inflammasome is detrimental to the regulation of the intestinal microbiota and exacerbates diseases. Consequently, further research investigating the mechanisms between intestinal flora and the NLRP3 inflammasome is warranted, as this could contribute to a deeper understanding of the interplay between intestinal flora and the genesis of inflammatory diseases and may provide new insights for the development of relevant therapeutic strategies.

### 3.5 Keywords analysis

**FIGURE 6:**
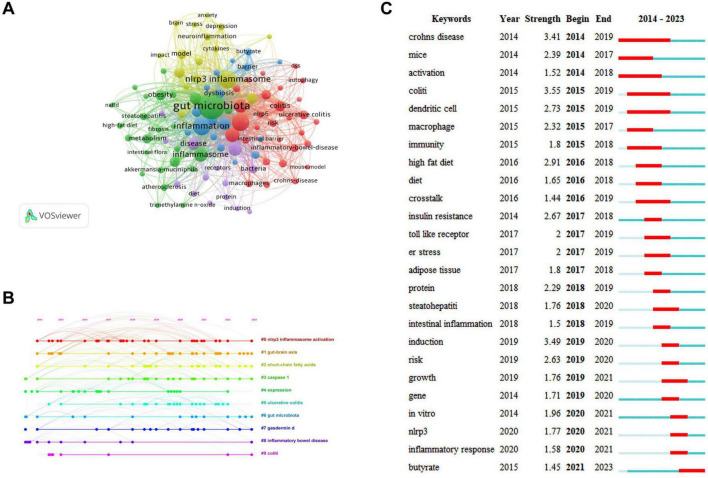
Keywords analysis. **(A)** Network visualization of keywords. **(B)** Timeline distribution of keywords. **(C)** Top 25 keywords with the strongest citation bursts.

Keywords are the core and essence of a paper, and through a co-occurrence analysis of keywords, hotspots in a certain research field can be revealed. A total of 2,277 keywords were extracted for analysis. For the 122 keywords that occurred eight or more times, VOSviewer and CiteSpace software were jointly used to visualize the keywords (Figure 6A). The larger the node size, the higher is the frequency of keyword occurrence, and this is more representative of field hotspots. Node connections represent the strength of association, with thicker connections indicating a greater number of times that both appear in the same article. Node colors represents different clusters of research themes. Five clusters were identified in this study. The red cluster is primarily associated with inflammatory bowel diseases (colitis and Crohn’s disease), the immune system, and NLRP6 inflammasome. The green cluster is primarily related to inflammatory diseases (atherosclerosis, insulin resistance, steatohepatitis, and obesity), gut microbiota, fat metabolism, and the NLRP3 inflammasome. The blue cluster is primarily associated with gut microbiota metabolites (short-chain fatty acids, butyrate), intestinal barrier damage, and the immune system. The yellow cluster is primarily related to the microbiota-gut-brain axis, neuro-inflammation, and NLRP3 inflammasome, and the purple cluster is associated with the gut-liver axis and bacteria.

The keyword network visualization of VOSviewer that can intuitively display hot topics in a field does not consider temporal factors. The CiteSpace timeline diagram arranges keywords in a temporal sequence to provide a clear representation of the hot topic distribution over time periods (Wang J. et al., 2023). To predict the hot topics in the field of research under investigation more accurately, we analyzed the changes in research hotspots for keywords from 2014 to 2023 using the timeline diagram of CiteSpace (Figure 6B). The main keywords were categorized into 10 major clusters: “#0 NLRP3 inflammasome activation,” “#1 gut-brain axis,” “#2 short-chain fatty acids,” “#3 caspase-1,” “#4 expression,” “#5 ulcerative colitis,” “#6 gut microbiota,” “#7 gasdermind,” “#8 inflammatory bowel disease,” and “#9 colitis.” The dots appearing on each timeline represented the high-frequency keyword nodes appearing in that cluster. By combining the keywords in each timeline, the clusters were classified into four categories that included gut microbiota (#1, #2, #4, and #6), NLRP3 inflammasome (#0 and #4), pyroptosis (#3 and #7), and inflammatory diseases (#4, #5, #8, and #9). Through the above analysis, we determined that in the field of research examining gut microbiota and inflammation-induced pyroptosis over the past decade, researchers have primarily focused on the gut microbiota, NLRP3 inflammasome, pyroptosis pathways, and inflammatory diseases. Furthermore, almost all clusters are expected to receive attention in 2023.

To gain a clearer understanding of the specifics of the keywords, Table 6 ranks the 15 most frequently used keywords in descending order of frequency. The keyword with the highest frequency was “gut microbiota,” and this was followed by “NLRP3 inflammasome,” “inflammation,” and “activation” that exhibited frequencies exceeding 100, indicating that they received considerable attention from researchers in past related studies.

**TABLE 6:** Top 15 high-frequency keywords.

Number	Keywords	Occurrences
1	Gut microbiota	267
2	NLRP3 inflammasome	121
3	Inflammation	119
4	Activation	110
5	Microbiota	76
6	Inflammasome	71
7	Mice	64
8	Expression	62
9	NLRP3	54
10	Disease	51
11	Cells	50
12	Oxidative stress	46
13	Colitis	45
14	Mechanisms	45
15	Chain fatty acids	43

The red and blue lines depict the citation burst from 2012 to 2023, and the length of the red line indicates the duration of the citation burst in Figure 6C. The analysis of the burst keywords map revealed that the keyword “colitis” possessed the highest burst intensity, consistently experiencing a high-burst state from 2015 to 2019, before leveling off. The other two keywords with a burst intensity greater than 3 were “Crohn’s disease” and “induction.” These high-burst keywords indicate that over time, researchers have placed greater emphasis on inflammatory bowel diseases. Currently, the keyword experiencing a burst state in the field of research is “butyrate,” indicating that primary focus of researchers is on butyrate, a metabolite of the gut microbiota.

### 3.6 Future prospects

Research directions associated with frequently co-cited literature and trending keywords are expected to remain focal points for researchers in this field. Analysis of these co-citations and keywords can provide insights into future research trends (Wang S. et al., 2023). Among the highly cited references, seven articles remained in a citation burst state in 2023. These studies have primarily focused on the mechanisms underlying the interactions between the gut microbiota, NLRP3 inflammasomes, and inflammatory diseases. Next, in a timeline of keywords, research within this domain over the past decade has primarily focused on the gut microbiota, NLRP3 inflammasome, pyroptosis, and inflammatory diseases, and these topics will continue to garner interest in 2023. Finally, in the burst word plot, only butyrate remains in a burst state in 2023. In summary, the future research focus in this field is likely to be focused on the mechanism of action among butyrate, pyroptosis mediated by NLRP3 inflammasome, and inflammatory diseases.

**FIGURE 7:**
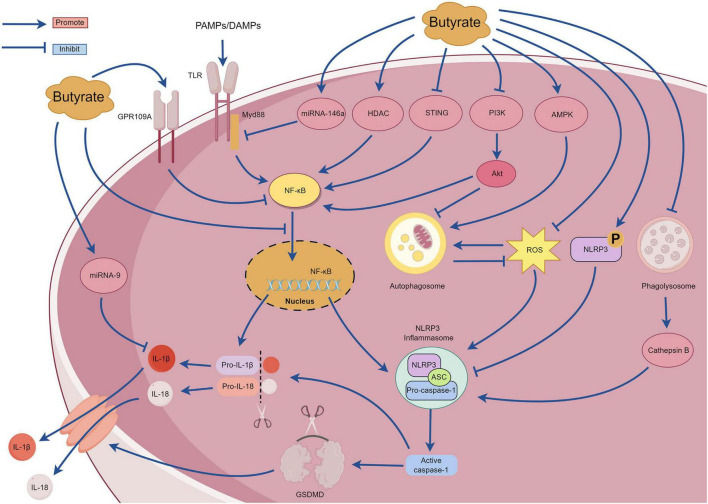
The mechanism of pyroptosis mediated by NLRP3 inflammasome regulated by butyrate.

SCFAs are the primary metabolites produced by the breakdown of dietary fiber by the gut microbiota and predominantly comprise acetic, propionic, and butyric acids (in a ratio of 3:1:1). These metabolites provide energy to the intestine, regulate the diversity of the gut microbiota, and enhance intestinal microbial barrier (Li, 2022). With continued in-depth studies investigating SCFAs, butyrate has garnered attention due to its extensive pharmacological activity (Dang et al., 2021). Butyrate exhibits promising therapeutic effects for treating various inflammatory diseases, including inflammatory bowel disease, pancreatitis, allergic rhinitis, and sepsis (Vernia et al., 2000; Zhang et al., 2007; Kanika et al., 2015; Wang et al., 2016). An increasing number of recent studies have examined the mechanisms underlying the action of butyrate, most of which involve NLRP3 inflammasome-mediated pyroptosis. However, there are no comprehensive reviews examining the relationship between butyrate and NLRP3 inflammasome-mediated pyroptosis. The section summarizes the mechanisms of interaction between butyrate and the NLRP3 inflammasome-mediated pyroptosis to provide a reference for researchers (Figure 7).

Under high-fiber diet conditions, acetate can promote Ca^2+^ mobilization and K^+^ efflux through G protein-coupled receptors such as GPR109A and GPR43, thereby activating the assembly of the NLRP3 inflammasome (Macia et al., 2015). However, Pan et al. (2019) observed that butyrate inhibited NF-κB p65 phosphorylation and NLRP3 inflammasome activation by upregulating the expression of GPR109A rather than upregulating expression of GPR41. Additionally, Wu et al. (2021) reported that butyrate can upregulate the expression of GPR109A. Subsequently, butyrate was unable to inhibit the activation of NLRP3 inflammasome in GPR109A^/^ mice, indicating that butyrate promotes the expression of GPR109A. This in turn inhibits the activation of the downstream NLRP3 inflammasome. Therefore, the mechanism of butyrate regulation through the GPR109A receptor requires further investigation. Tian et al. (2023) discovered that exogenous supplementation with butyrate in mice can reduce the expression of the p65 subunit of NF-κB, p-NF-κB inhibitor α (p-IκBα), and proteins related to NLRP3-mediated pyroptosis (NLRP3, IL-1β, caspase-1, and GSDMD) and effectively ameliorate renal fibrosis in mice. The mechanism involves inhibiting pyroptosis mediated by the NLRP3 inflammasome by regulating the stimulator of interferon genes (STING)/NF-κB p65 pathway. However, further studies are required to elucidate the underlying mechanisms. Gu et al. (2019) reported that sodium butyrate can inhibit the nuclear translocation of NF-κB and the caspase-1-GSDMD pyroptosis pathway, thereby protecting endothelial cells from pyroptosis induced by high glucose levels. Hyperglycemia can activate the phosphoinositide 3-kinase (PI3K)/protein kinase B (Akt) signaling pathway, and this subsequently upregulates IκBα phosphorylation and NF-κB signaling activation (Pan et al., 2020). MicroRNA-9 and microRNA-146a are also crucial for the regulation of the NF-κB pathway (Wu et al., 2018). Clinical studies have determined that after taking butyrate at a dose of 600 mg/day for several days, patients with type 2 diabetes exhibit increased expression of microRNA-9 and microRNA-146a in their plasma that negatively correlate with the expression of pyroptosis-related mRNAs (TLR2, TLR-4, NF-κB, caspase-1, NLRP3, IL-1β, and IL-18) in peripheral blood mononuclear cells (Roshanravan et al., 2020). These findings suggest that butyrate may affect the pyroptosis signaling pathway mediated by the (NF-κB)-NLRP3 inflammasome by regulating the expression of microRNA-9 and microRNA-146a. However, the specific regulatory mechanisms remain unclear and require further investigation. Although these results suggest that butyrate possesses multiple mechanisms for regulating the NF-κB signaling pathway and can improve inflammation, other studies have demonstrated that butyrate can exacerbate neuroinflammation and enteric inflammation in mice with Parkinson’s disease induced by 1-methyl-4-phenyl-1,2,3,6-tetrahydropyridine (MPTP), and this mechanism is not related to the NF-κB/MyD88/TNF-α signaling pathway. Despite substantial impairment of the intestinal barrier in mice with Parkinson’s, an increase in inflammatory cytokines and NLRP3 mRNA expression was not detected in the colonic tissues (Qiao et al., 2020). Further investigations are warranted to elucidate the underlying mechanisms. Yuan et al. (2018) discovered that butyrate substantially inhibits the formation and activation of the NLRP3 inflammasome induced by cholesterol in endothelial cells, reducing the production of inflammatory factors and O_2_. This mechanism involves blocking lipid valve redox signaling platforms. Histone deacetylase can regulate the expression of inflammatory factors by inhibiting the acetylation of NF-κB (Ashburner et al., 2001). Jiang et al. (2020) observed that sodium butyrate can improve inflammation in macrophage cells by reducing the expression of histone deacetylase and NLRP3 proteins and by inhibiting the phosphorylation of IκBα and NF-κB p65 subunit. Cleophas et al. (2016) discovered that butyrate specifically inhibited class I histone deacetylases, thereby inhibiting the production of inflammatory factors induced by uric acid sodium crystals in peripheral blood mononuclear cells. However, the mechanism by which butyrate affects cholesterol-induced lysosomal damage and subsequently activates the NLRP3 inflammasome requires further investigation. Phosphorylation of the Ser295 site of NLRP3 is important for activating the NLRP3 inflammasome (Huang and Zhou, 2021). Bai et al. (2023) reported that sodium butyrate can promote phosphorylation of the NLRP3 protein at the Ser295 site and inhibits NLRP3 inflammasome-mediated pyroptosis. Additionally, sodium butyrate can inhibit the NF-κB pathway, reduce autophagy stress, and finally reduce kidney injury and fibrosis. The excessive production of ROS is also closely related to the activation of NF-κB signaling pathway and the activation of NLRP3 inflammasome. ROS can promote the phosphorylation of IκBα and then promote the formation of NF-κB dimers. Additionally, ROS can regulate the DNA-binding activity of NF-κB in the nucleus, and then promote the upregulation of NLRP3 and pro-IL-1β (Li W. et al., 2022). Shu et al. (2022) compared the anti-inflammatory effects of sodium acetate, sodium butyrate and sodium propionate on *Acinetobacter baumannii*-induced THP-1 cells. Sodium butyrate can inhibit the expression of NLRP3 protein, caspase-1 and GSDMD, inhibit the translocation of NF-κB p65 to the nucleus, activate cell autophagy, and substantially downregulate the expression of ROS, TLR-2, IL-1β, and IFN-γ. Zhang Y. et al. (2023) observed that the sodium acetate, sodium propionate, and sodium butyrate could inhibit macrophage inflammation by inhibiting the ROS/NF-κB/NLRP3 signaling pathway and regulating glycerophospholipid and sphingolipid metabolism. Singh et al. (2023) reported that butyrate promoted the production of antimicrobial peptides in the infected retinas of mice and activated autophagy by enhancing adenosine monophosphate-activated protein kinase (AMPK), thereby reducing bacteria-induced intraocular inflammation and the production of NLRP3 protein and caspase-1. However, after treatment with an NLRP3 inhibitor, butyrate improved the corneal state and inhibited the release of inflammatory mediators, suggesting that butyrate exerts its anti-inflammatory effects through NLRP3-independent pathways. Sodium butyrate could also activate the AMPK pathway to reduce the expression of ROS, NLRP3 protein, caspase-1, and IL-1β, and it could improve mitochondrial autophagy function and intestinal epithelial barrier function (Li X. et al., 2022). However, when an AMPK inhibitor or mitochondrial autophagy inhibitor was used, the protective effect of sodium butyrate on mitochondria and the intestinal epithelial barrier was weakened. Yan et al. (2022) observed that in a glycosylation product-induced macrophage inflammatory injury model, sodium butyrate could inhibit the entry of NF-κB/p65 protein into the nucleus and the activation of NLRP3 inflammasome by blocking the PI3K/Akt/NF-κB pathway. Sodium butyrate also reduces intracellular ROS levels and increases malondialdehyde (MDA) and superoxide dismutase (SOD) to reduce oxidative stress injury. This study determined that sodium butyrate may inhibit autophagy by inhibiting the PI3K/Akt pathway; however, the relationship between this pathway and autophagy requires further investigation.

## 4 Discussion

Élie Metchnikoff proposed the concept of the gut microbiota in the early 20th century and analyzed its relationship with the immune system, highlighting its role in the pathogenesis of inflammatory diseases (Mackowiak, 2013). In the year 2010, a comprehensive study conducted by the Chinese Academy of Sciences employed high-throughput sequencing methodologies to establish the inaugural human gut microbial gene catalog. This investigation revealed in excess of 3 million microbial genes residing within the gut, thereby markedly enhancing our comprehension of the diversity inherent in the human gut microbiota (Qin et al., 2010). This research outcome has accelerated the systematic study of gut microbiota. Pyroptosis was initially introduced in 2001 by s research group headed by the American scientist Brad T. Cookson, representing a noteworthy progression in the comprehension of inflammatory pathologies (Cookson and Brennan, 2001). Investigating the interaction between gut microbiota and pyroptosis is essential for clarifying the fundamental mechanisms that proper the advancement of inflammatory disorders.

In this study, we analyzed 520 articles from 41 countries, 887 institutions, and 4,212 authors published in 245 journals within the research field. Over the past decade, the number of publications in this field has steadily increased. Although citations have declined in recent years, the field continues to receive widespread attention from researchers. Our findings, combined with the contributions of China and the United States in this field, reveal that China ranks as the leading country in publication volume, whereas the United States leads in citation impact, underscoring the substantial influence of these two nations in advancing this area of research. All of the top 10 core research institutions by publication volume are based in China, indicating the widespread attention and in-depth research by Chinese institutions in this field. It is of considerable importance to note Eicke Latz, affiliated with the University of Bonn, possesses the preeminent total citation count and average citation rate, thereby emphasizing his considerable academic impact within the discipline. His scholarly endeavors predominantly concentrate on elucidating the mechanisms that govern the innate immune system and its involvement in inflammatory processes (Christ et al., 2018; Wang et al., 2024). These research pursuits have established essential theoretical frameworks for the innovation of novel immunotherapeutic approaches and disease management strategies.

Inflammasomes represent intricate protein assemblies that identify cellular distress or the incursion of pathogens, thereby initiating the activation of caspases, which subsequently facilitate the process pyroptosis (Huang et al., 2021). In analysis of the co-cited literature revealed considerable attention to the relationship between the NLRP3 and NLRP6 inflammasomes and gut microbiota. NLRP3 inflammasome plays a critical role in the pathogenesis of various diseases, including cardiovascular diseases, metabolic disorders, and neurodegenerative diseases. Inhibiting its activation can significantly improve disease progression (Mangan et al., 2018). Moreover, recent findings have underscored the emerging role of the NLRP6 inflammasome in regulating infection disease, colitis, and cancer (Barnett et al., 2023). Keyword analysis reveals that butyrate is currently in an explosive phase of research, indicating that its role in regulating inflammasome-mediated pyroptosis has become a major focus in this field. A comprehensive analysis of these co-citations and keywords suggests that future research in this field may explore the mechanisms underlying the interactions between butyrate, NLRP3 inflammasome-mediated pyroptosis, and inflammatory diseases. Butyrate plays an important role in the regulation of diseases, and maintaining a diet with high dietary fiber content will help balance intestinal flora and physical health (Yao et al., 2022). Currently, the mechanism by which butyrate activates or inhibits the NLRP3 inflammasome is unclear; however, it is established that NLRP3 inflammasome-mediated pyroptosis plays an important role in improving or aggravating the occurrence of diseases. Further experimental studies investigating butyrate, NLRP3 inflammasome-mediated pyroptosis, and inflammatory diseases are required to understand the beneficial effects of butyrate on inflammatory diseases and promote the development and application of butyrate as a therapy.

To the best of our knowledge, this is the first bibliometric analysis of the literature published in the field of intestinal flora and inflammasome-mediated pyroptosis in the past 10 years. This analysis provides valuable insights for researchers interested in this area. We have employed a variety of reliable bibliometric tools to ensure that our data is as accurate and objective as possible. Additionally, compared to traditional reviews, bibliometric analysis offers the advantage of not only summarizing the development of the research field but also predicting future research trends, which can better guide future investigations in this domain. Our advantage primarily lies in systematically articulating and mapping the trajectory of research hotspots, followed by a literature review based on data analysis. However, this study possessed some limitations. First, as bibliometric visual analysis software can only analyze data from a single database, this study selected only the most commonly used Web of Science Core Collection that can also be selected from PubMed, Scopus, ScienceDirect, and other databases. Second, in the keyword combination, the direct combination of “intestinal flora,” “inflammasome,” and “pyroptosis” was rare, so the inflammasome and pyroptosis were connected by the word “OR” instead of “AND.” Third, when collecting and analyzing data, there will inevitably be subjectivity of human factors, and this will lead to certain deviations from the real situation in literature screening and statistical analysis.

## 5 Conclusion

The increasing interest among researchers regarding the role of the gut microbiota and inflammasome-mediated pyroptosis has been notable, yet a bibliometric analysis of the related literature remains absent. This study employs bibliometric methods to review the research trends and emerging topics in this field over the past decade, as well as potential future directions. For much of this period, the focus has been on elucidating the mechanisms linking the gut microbiota with NLRP3 or NLRP6 inflammasome-mediated pyroptosis. With continued advancements in the understanding of these mechanisms, future research in this domain is likely to explore the relationship between butyrate, NLRP3 inflammasome-mediated pyroptosis, and inflammatory diseases. However, further experimental investigations are still required to fully elucidate the underlying mechanisms of these interactions.

## Data Availability

The original contributions presented in this study are included in this article/supplementary material, further inquiries can be directed to the corresponding authors.

## Footnotes

1
https://www.vosviewer.com

2
https://citespace.podia.com

3
https://www.graphica.app

4
https://www.graphpad.com
